# The prevalence and sociodemographic determinants of tobacco and nicotine use among students in healthcare disciplines in Saudi Arabian universities: a cross-sectional survey

**DOI:** 10.3389/fpubh.2024.1348370

**Published:** 2024-03-07

**Authors:** Abdulelah M. Aldhahir, Rayan A. Siraj, Abdullah A. Alqarni, Jaber S. Alqahtani, Mohammed M. Alyami, Mansour S. Majrshi, Hassan Alwafi, Mohammed M. Alqahtani, Sarah S. Monshi, Abdulmohsen H. Al-Zalabani, Abdullah M. Alanazi

**Affiliations:** ^1^Respiratory Therapy Department, Faculty of Applied Medical Sciences, Jazan University, Jazan, Saudi Arabia; ^2^Department of Respiratory Care, College of Applied Medical Sciences, King Faisal University, Al-Ahsa, Saudi Arabia; ^3^Department of Respiratory Therapy, Faculty of Medical Rehabilitation Sciences, King Abdulaziz University, Jeddah, Saudi Arabia; ^4^Respiratory Therapy Unit, King Abdulaziz University Hospital, Jeddah, Saudi Arabia; ^5^Department of Respiratory Care, Prince Sultan Military College of Health Sciences, Dammam, Saudi Arabia; ^6^Respiratory Therapy Department, Batterjee Medical College, Khamis Mushait, Saudi Arabia; ^7^National Heart and Lung Institute, Imperial College London, London, United Kingdom; ^8^Respiratory Medicine, Royal Brompton Hospital, London, United Kingdom; ^9^Faculty of Medicine, Umm Al-Qura University, Mecca, Saudi Arabia; ^10^Department of Respiratory Therapy, College of Applied Medical Sciences, King Saud bin Abdulaziz University for Health Sciences, Riyadh, Saudi Arabia; ^11^King Abdullah International Medical Research Center, Riyadh, Saudi Arabia; ^12^Department of Health Administration and Hospitals, College of Public Health and Health Informatics, Umm Al-Qura University, Makkah, Saudi Arabia; ^13^Department of Family and Community Medicine, College of Medicine, Taibah University, Madinah, Saudi Arabia

**Keywords:** smoking, tobacco, nicotine, healthcare, students

## Abstract

**Background:**

Tobacco smoking is one of the most significant health-related problems worldwide. However, the prevalence and sociodemographic determinants of tobacco and nicotine use among students on healthcare courses in Saudi Arabia are limited.

**Methods:**

This cross-sectional study used a questionnaire that was distributed via SurveyMonkey between November 2022 and June 2023 to all student universities offering healthcare courses. Data are presented as frequency and percentages. The associated sociodemographic factors with current tobacco and nicotine use were subjected to logistic regression.

**Results:**

Overall, 5,012, of whom 3,872 (77.25%) were males, responded to the online survey. The prevalence of current tobacco and nicotine use was 3,553 (71%). The majority of users used electronic nicotine delivery systems (1,622: 32.36%). The current use of cigarettes (AOR: 1.75 (1.42 to 2.15)), e-cigarettes (AOR: 1.17 (1.01 to 1.37)), and smokeless tobacco substances (AOR: 1.35 (1.02 to 1.90)) were more pronounced among males compared to female students. However, the current use of a hookah was less among males (AOR: 0.74 (95% CI: 61 to 0.91)). Smoking cigarettes was practiced less among students living in other regions compared to the Central Region. However, smokeless tobacco substances seem to be more prevalent in the Western, Southern, and Northern Regions, (AOR: 1.57 (95% CI: 1.09 to 2.26)), (AOR: 1.43 (95% CI: 1.04 to 1.95)), and (AOR: 1.57 (95% CI: 1.09 to 2.26)), respectively.

**Conclusion:**

Smoking is prevalent among students in the healthcare disciplines, with electronic nicotine delivery systems being the most used product. Several sociodemographic factors were associated with higher tobacco or nicotine use.

## 1 Introduction

Tobacco smoking is one of the most significant health-related problems worldwide. It poses a considerable risk for various diseases, including different types of cancers, cardiovascular diseases, and pulmonary diseases (1–3). Further, reports indicate that tobacco use has led to a high death rate globally: In 2019, tobacco was implicated in approximately 8.71 million fatalities worldwide and contributed to around 229.77 million disability-adjusted life years on a global scale (4, 5). Moreover, the use of tobacco is associated with a decrease in physical and social abilities, diminished energy levels, poor general health, and compromised mental well-being (6). Smoking goes beyond being a health concern; it may also pose a hurdle to academic success. For instance, a study revealed that smokers had significantly lower GPAs, higher absenteeism rates, and a greater number of academic warnings compared to non-smokers (7).

Tobacco use, in all its forms, has become more pervasive among college students. For instance, one study has indicated that the prevalence of tobacco use among nursing students is pervasive (8). The research indicates that 12.4% of medical students actively smoke, while almost 40% of these students are exposed to secondhand smoke. When breaking down the data by gender, they reveal that 18.6% of male medical students and 5.9% of female medical students are active smokers (9). Smoking was found to be most common in European countries, with 20% of medical students and 40% of dental students reported to be smokers. Similarly, in the Americas, the rates ranged from 13% among pharmacy students to 23% among dental students. Additionally, the use of other tobacco products was notably high in Eastern Mediterranean countries (10–23%) and in Europe (7–13%) (10). It has also been reported in one study that around 24% of students indicated they use e-cigarettes daily and around 50% of these students mentioned that they had used an e-cigarette in the last 30 days (11). Another study has revealed that around 27% of college students reported they used e-cigarettes (12). Interestingly, in the Middle East, specifically in Saudi Arabia, there is a notable lack of national-level research regarding the prevalence and sociodemographic determinants of tobacco and nicotine use among students specializing in healthcare. This gap in the research highlights the need for a national survey in Saudi Arabia to understand better the sociodemographic factors and theoretical frameworks that influence tobacco and nicotine use in this particular student group.

The goal of this study is to assess the prevalence and sociodemographic determinants of tobacco and nicotine use among healthcare specialty students in Saudi Arabia through a national survey. Our study may pave the way toward a better understanding of how sociodemographic characteristics are associated with nicotine/tobacco behaviors among Saudi Arabian healthcare students. The findings could also support interventions aimed at ceasing or preventing nicotine and tobacco use by healthcare students here.

## 2 Methods

### 2.1 Study design and instrument

This cross-sectional study used a questionnaire that was developed to meet the study objectives. The questionnaire consisted of six items in two sections as follow:

Section one consisted of five questions including age, gender, geographical region profession and academic level.

Section Two consisted of one question regarding tobacco and nicotine use in the past 30 days.

The questionnaire was piloted and evaluated by 10 participants from the target population to ensure the comprehensibility and clarity of the questions, as well as the answers.

### 2.2 Data collection and sampling

The questionnaire was available and distributed via the SurveyMonkey platform from 1st of December 2022 to 30th of June 2023 to all universities in Saudi Arabia. Each data collector was responsible for distributing the survey to several universities via professional groups on social media platforms or by contacting the assigned university. A convenience sampling strategy was utilized and students, male and female, who currently study the healthcare disciplines at a university in Saudi Arabia were the main target and were invited to participate. Before starting the survey, written consent was insured for all participants and voluntary participation was confirmed by asking participants if they were happy to be in the study and they received information about the study, as well as contact information for the chief researcher. Repetitive and duplicate responses were avoided by only allowing the participants to fill out the survey link once. The estimated time to complete the questionnaire was 3 minutes.

### 2.3 Data analysis

The data were analyzed using the Statistical Package for Social Sciences (SPSS software, Version 29). Frequency and percentages were used to summarize demographic characteristics. Age was calculated using mean and standard deviation. The associated sociodemographic factors with current tobacco and nicotine use were subjected to multivariate logistic regression analysis. A *p* value of <0.05 was considered statistically significant.

### 2.4 Ethical approval

The Bioethics and Research Committee at Jazan University, Saudi Arabia approved this study with reference number REC-44/04/363.

## 3 Results

A total of 5,012 participants were included in the analyses. The mean (±SD) age of the study population was 21 (±2) years, and there were more males (3,872, 77.25%) than females. The vast majority of participants were from the Southern Region of Saudi Arabia (2,089, 41.68%), followed by the Central Region (886, 17.68%) (see Table 1). Of students across different healthcare disciplines, nursing students accounted for 1,078 (21.5%), followed by nutrition and respiratory therapy students, 750 (15%) and 614 (12%), respectively. Third-year (1,513, 30%) and fourth-year (1,231, 25%) students accounted for more than half of the population. Full details of the demographic data are presented in Table 1.

**Table 1:** Demographic data of study participants (*n* = 5,012).

Variable	n (%) or Mean (SD)
Age, years (Mean (SD))	21 (±2)
Gender (male %)	3,872 (77.25%)
Geographical Region, n (%)	
Southern Region	2,089 (41.68%)
Central Region	886 (17.68%)
Western Region	824 (16.44%)
Eastern Region	646 (12.89%)
Northern Region	567 (11.31%)
Profession, n (%)	
Nursing	1,078 (21.51%)
Nutrition	750 (14.96%)
Respiratory Therapy	614 (12.25%)
Dentistry	524 (10.45%)
Medicine	492 (9.82%)
Physiotherapy	447 (8.92%)
Public health	412 (8.22%)
Pharmacy	314 (6.26%)
Medical laboratory	250 (4.99%)
Emergency medical services	131 (2.61%)
Academic level, n (%)	
1^st^ year	465 (9.28%)
2^nd^ year	1,206 (24.06%)
3^rd^ year	1,513 (30.19%)
4^th^ year	1,231 (24.56%)
5^th^ year	331 (6.60%)
6^th^ year	125 (2.49%)
Internship	141 (2.81%)
Current use, n (%)	
Electronic nicotine delivery system (e-cigarettes, e-hookah, vaping)	1,622 (32.36%)
Tobacco Cigarettes	812 (16.20%)
Hookah (Waterpipe, Muʽassel, Jurak)	710 (14.17%)
Smokeless Tobacco (Shammah)	409 (8.16%)
Any product use	3,553 (70.89%)
Never used	1,459 (29.11%)

Data are presented as n (%) or mean SD. GPA, Grade Point Average. Current smoker: someone who has smoked within the past 30 days.

### 3.1 Prevalence of current tobacco and nicotine users

The prevalence of current any product (tobacco and nicotine) use among students in the healthcare disciplines was 3,553 (70.89%). The majority of users used electronic nicotine delivery systems (1,622, 32.36%), followed by cigarettes (812, 16.20%) and hookahs (710, 14.17%). See Table 1.

### 3.2 Demographic factors associated with current use of tobacco and nicotine

The current use of tobacco and nicotine substances (e.g., cigarettes, e-cigarettes, smokeless tobacco, and any product use) was more prevalent among males compared to female students. See Table 2. Meanwhile, the current use of hookahs was less among males (AO.R: 0.74 (95% CI: 61 to 0.91)). Smoking cigarettes were less among students living in other regions compared to the Central Region. Smokeless tobacco substances seemed to be more prevalent in the Western, Southern and Northern Regions (AO.R: 1.57 (95% CI: 1.09 to 2.26)), (AO.R: 1.43 (95% CI: 1.04 to 1.95)), and (AO.R: 1.57 (95% CI: 1.09 to 2.26)), respectively. Use of cigarettes, hookahs, and smokeless tobacco substances did not differ across academic levels, whereas the current use of e-cigarettes and any product use were more common among students in their mid and advanced academic stages compared to those in their 1st year.

**Table 2:** Multivariate logistic regression models of the association between demographic variables (independent) and current tobacco and nicotine use (dependent).

Descriptor	AO.R (95% CI) Cigarettes smoking	AO.R (95% CI) Hookah	AO.R (95% CI) E-cigarettes	AO.R (95% CI) Smokeless tobacco	AO.R (95% CI) Any product use
Gender					
Female	1	1	1	1	1
Male	1.75 (1.42 to 2.15)	0.74 (0.61 to 0.91)	1.17 (1.01 to 1.37)	1.35 (1.02 to 1.90)	1.56 (1.34 to 1.82)
Region					
Central	1	1	1	1	1
Eastern	0.60 (0.45 to 0.79)	1.12 (0.85 to 1.48)	1.12 (0.90 to 1.40)	1.27 (0.85 to 1.90)	0.91 (0.72 to 1.17)
Western	0.59 (0.45 to 0.77)	1.02 (0.77 to 1.33)	1.11 (0.91 to 1.37)	1.57 (1.09 to 2.26)	0.93 (0.73 to 1.17)
Southern	0.69 (0.56 to 0.85)	0.80 (0.64 to 1.01)	0.85 (0.71 to 1.01)	1.43 (1.04 to 1.95)	0.63 (0.52 to 0.76)
Northern	0.78 (0.59 to 1.03)	0.53 (0.37 to 0.76)	0.92 (0.73 to 1.16)	1.57 (1.09 to 2.26)	0.57 (0.45 to 0.73)
Profession					
Dentistry	1	1	1	1	1
Medicine	0.67 (0.44 to 1.03)	2.55 (1.71 to 3.80)	0.60 (0.46 to 0.79)	1.55 (0.98 to 2.44)	0.92 (0.69 to 1.22)
Nursing	1.96 (1.43 to 2.68)	2.22 (1.54 to 3.19)	0.64 (0.51 to 0.80)	1.31 (0.86 to 1.99)	1.58 (1.23 to 2.02)
Nutrition	1.80 (1.29 to 2.52)	1.37 (0.92 to 2.03)	0.52 (0.41 to 0.66)	0.96 (0.61 to 1.51)	0.79 (0.61 to 1.01)
Pharmacy	2.18 (1.48 to 3.19)	1.53 (0.95 to 2.46)	0.41 (0.30 to 0.57)	0.99 (0.55 to 1.79)	0.79 (0.58 to 1.08)
EMS	0.42 (0.18 to 0.95)	3.47 (2.05 to 5.87)	0.66 (0.44 to 0.99)	1.58 (0.83 to 3.01)	1.26 (0.77 to 2.05)
Medical lab	1.03 (0.65 to 1.64)	2.17 (1.35 to 3.49)	0.66 (0.44 to 0.99)	1.87 (1.12 to 3.11)	0.72 (0.52 to 1.01)
PT	2.11 (1.48 to 3.01)	1.56 (1.01 to 2.40)	0.47 (0.35 to 0.62)	0.64 (0.36 to 1.13)	0.80 (0.61 to 1.06)
PH	0.67 (0.43 to 1.04)	1.59 (1.02 to 2.47)	1.01 (0.77 to 1.32)	1.67 (1.05 to 2.66)	1.25 (0.93 to 1.69)
RT	1.24 (0.87 to 1.77)	2.61 (1.76 to 3.85)	0.60 (0.46 to 0.77)	0.97 (0.60 to 1/56)	1.01 (0.77 to 1.32)
Academic level					
1st year	1	1	1	1	1
2nd year	1.18 (0.88 to 1.58)	0.80 (0.57 to 1.08)	1.22 (0.94 to 1.58)	0.64 (0.44 to 0.92)	0.97 (0.76 to 1.23)
3rd year	1.05 (0.79 to 1.40)	0.66 (0.49 to 0.89)	1.63 (1.27 to 2.09)	0.59 (0.41 to 0.85)	1.04 (0.82 to 1.31)
4th year	0.93 (0.68 to 1.26)	0.79 (0.58 to 1.08)	2.01 (1.55 to 2.59)	0.61 (0.41 to 0.89)	1.35 (1.05 to 1.73)
5th year	0.88 (0.58 to 1.34)	1.06 (0.72 to 1.57)	2.46 (1.78 to 3.39)	0.84 (0.51 to 1.37)	2.58 (1.79 to 3.72)
6th year	0.84 (0.45 to 1.58)	0.93 (0.54 to 1.61)	2.18 (1.41 to 3.36)	1.47 (0.81 to 1.67)	2.76 (1.61 to 4.72)
Internship	0.63 (0.33 to 1.22)	0.37 (0.19 to 0.72)	1.75 (1.15 to 2.67)	1.12 (0.62 to 2.01)	0.91 (0.60 to 1.38)

EMS, emergency medical services; PT, RT, Physiotherapy; PH, Public health; Respiratory Therapy; AOR, adjusted odd ratio; E-NDS, Electronic nicotine delivery system.

## 4 Discussion

This national survey assessed the current use of tobacco and nicotine use among students in the healthcare disciplines in Saudi Arabia and examined the sociodemographic factors associated with tobacco and nicotine among this group. The study revealed that 71% of students were current tobacco/nicotine users, with most of them being males in advanced academic years and internship years. Electronic nicotine delivery systems were the most commonly used product. The study also indicated that tobacco/nicotine use among students in health colleges was associated with several sociodemographic factors, including gender, region of residency, specialty, academic years, and tobacco/nicotine types.

The findings of this study are similar to previous studies conducted in Saudi Arabia and other countries. In Saudi Arabia, tobacco use is prevalent among college students (13). One study indicated that 46.4% of the 895 students reported initiation of tobacco use when entering university (14). By comparing our findings with several recent studies, it can be seen that there is an increase in using e-cigarettes among students in health colleges, in particular (15). Al-Regaiey et al. reported that of 364 students studying in the health disciplines, the prevalence of vaping was 29.9% (12). The issue of tobacco/nicotine use among college students has also been observed in several GCC countries. Aligned with our findings, studies conducted in Bahrain, Kuwait, Qatar, and UAE found that tobacco use is prevalent among college students, and the current use of tobacco was greater among male students compared to female students, and those who were in advanced academic years (16–19). These studies also observed e-cigarette use among college students (16–19). However, a further study has indicated that the prevalence of smoking has statistically decreased among college students who study on smoke-free campuses (20). This study supports the need for active enforcement of on-campus smoke-free policies and on-campus cessation support clinics.

In agreement with other studies, male students had significantly higher odds of lifetime and current use of cigarettes, smokeless tobacco, and other nicotine products (12). Although this study did not account for the dual use of tobacco products, the results showed a pattern of several tobacco products used among students, among them e-cigarettes. Using combustible tobacco along with other products, such as e-cigarettes, is common among youth (21, 22). Dual use of tobacco products is associated with higher odds of cardiovascular diseases and respiratory illness compared to the use of one tobacco product (23–25). Also, the tobacco use pattern found in this study has been observed globally and likely reflects sociocultural factors related to gender norms. However, it should be noted that the prevalence of tobacco use is increasing among women in Saudi Arabia and is expected to increase further in the coming decades (26). The observed and expected changes in tobacco use among women in Saudi Arabia could be related to cultural changes and having more women joining the workforce.

An interesting finding was the geographic variation observed, with lifetime and current cigarette use less common in the Eastern, Western, and Southern Regions compared to the Central Region. On the other hand, students in the Western, Southern, and Northern Regions are more likely to use smokeless tobacco compared to students in the Central Region. The underlying reasons for these regional differences are unclear but warrant further investigation. Tailored interventions and education for the high-risk regions may help reduce tobacco use. Several tobacco control interventions could be implemented to address the issue of tobacco use among students in health disciplines such as enforce in-campus smoke-free policy, run in-campus smoking cessation clinics, integrate intense tobacco control curriculum that helps in assessing scientific evidence and teaches critical skills to detect tobacco marketing activities, and engage students in developing and evaluating tobacco control measures in campus (27–29).

Monitoring the prevalence of tobacco/nicotine use among students in health disciplines and among health practitioners is essential because the patterns of tobacco use among these groups have significant implications. Tobacco use among students in health disciplines has been tracked across 70 countries through the Global Tobacco Surveillance System (GTSS). The GTSS revealed that the prevalence of smoking varied, with the highest rates observed in European countries (20% among medical students and 40% among dental students) and the Americas (ranging from 13% among pharmacy students to 23% among dental students). Other forms of tobacco use were more common in the Eastern Mediterranean (ranging from 10 to 23%). The GTSS provides insight into the beliefs of health students worldwide, as 70% of students believed in the role model status of health professionals and their responsibility in assisting patients with smoking cessation (10). Addtionally, lay people often have a positive perception of health practitioners and students in health fields. Health practitioners are assumed to be reliable sources of medical information and have valid information to share about health risk factors. Thus, tobacco/nicotine use among health practitioners and students in health colleges would send an implied and incorrect health message related to tobacco/nicotine use, lead to the normalization of tobacco/nicotine use, and make practitioners resistant to offering smoking cessation to patients (30).

Although students in health disciplines are more aware of the risks of tobacco use compared to others, a high number of tobacco users were found in this study. Several factors influence tobacco use among students, including tobacco marketing activities such as misleading information and tobacco imagery in media, gender norms, and social pressure (31–33). The increase in tobacco/nicotine use among students in health disciplines observed in our study and previous studies could also be explained by the fact that students at this age are in a transitional stage from school to university. The fact that university students usually enjoy less supervision from their parents and engage more with their peers and friends may lead to an increase in the risk of tobacco/nicotine uptake. In addition, the increase in tobacco/nicotine use among students in health colleges may be due to stress related to the difficulty of studying in this field since one of the motivations for tobacco use mentioned in a previous study was stress. Although this study did not collect data on students’ incomes, Saudi students who are studying in public universities receive a monthly stipend (S.R 1,000) and an additional amount (which varies across the health field) is added to students who are in their internship year. This income may increase the affordability and accessibility of tobacco products. Therefore, it is worth re-thinking the legal age of accessing tobacco and considering a policy to raise the minimum legal age for the sale of tobacco products from 18 to 21.

This study has revealed a finding of concern about the use of electronic cigarettes among the younger generations in Saudi Arabia. The popularity of electronic nicotine delivery systems is a growing public health concern as these products are often perceived, erroneously, as safe alternatives to traditional cigarettes. The present results suggest that there is marketing and promotion of electronic cigarettes among this group. This is not uncommon for a tobacco industry that uses tactics to market tobacco products and to target young people (34). A recent study that monitored the imagery of e-cigarettes in the media found an increase in the use of e-cigarettes in Arabic media and on-demand platforms, such as Netflix (35). In Saudi Arabia, Netflix is one of the most popular platforms among the youth. Portraying e-cigarettes appealingly and attractively in media scenes would promote the use of e-cigarettes and normalize this behavior among this group. Future studies should further examine the environmental factors affecting the use of electronic cigarettes among the younger generations. In addition, the implementation of tobacco control measures that ban tobacco/nicotine advertisement, promotion, and sponsorship, increase anti-tobacco awareness, and enforce smoke-free policies, especially on campuses, should be strengthened.

### 4.1 Strengths and limitations

This study has several strengths, including the large national sample size, the diverse geographic regions represented, and the inclusion of students across multiple healthcare disciplines. The comprehensive assessment of multiple nicotine and tobacco products provides a thorough picture of usage patterns.

Some limitations should be acknowledged. First, the self-reported survey design raises the possibility of recall or response bias. Second, we utilized convenience sampling, which may limit generalizability compared to a random sampling design. Also, the methodology employed for participant recruitment and the approach used for sample selection in this study impeded our ability to determine the response rate. However, the large sample across diverse universities enhances representativeness. Third, we did not assess details on frequency, history and start of use, dual-use, quantity or motivations underlying the use of different tobacco and nicotine products. Further research should aim to characterize start, change and end of use (tobacco cigarette, shisha, e-cigarettes, heated tobacco and heated nicotine products) before and while studying medicine, nursing or other healthcare.

## 5 Conclusion

This study provides valuable data to inform public health efforts aimed at reducing tobacco and nicotine use among future healthcare professionals in Saudi Arabia. Most healthcare discipline students showed high rates of use, underscoring the need for comprehensive interventions across nursing, medicine, dentistry, and other programs. Healthcare students who use nicotine and tobacco products themselves may be less likely to counsel their future patients about cessation (36). Integrating more robust tobacco cessation education into health sciences curricula could help address this issue.

## Data availability statement

The raw data supporting the conclusions of this article will be made available by the authors, without undue reservation.

## Ethics statement

The studies involving humans were approved by the Bioethics and Research Committee at Jazan University, Saudi Arabia approved this study with reference number REC-44/04/363. The studies were conducted in accordance with the local legislation and institutional requirements. The participants provided their written informed consent to participate in this study.

## Author contributions

AAld: Conceptualization, Data curation, Project administration, Writing – original draft, Writing – review & editing, Validation. RS: Formal analysis, Writing – original draft, Writing – review & editing. AAA: Conceptualization, Methodology, Writing – review & editing. JA: Resources, Supervision, Validation, Writing – review & editing. MAly: Investigation, Visualization, Writing – review & editing. MM: Investigation, Writing – review & editing. HA: Supervision, Writing – original draft, Writing – review & editing. MAlq: Data curation, Writing – original draft, Writing – review & editing. SM: Validation, Writing – original draft, Writing – review & editing. AA-Z: Writing – original draft, Writing – review & editing. AAla: Conceptualization, Validation, Writing – original draft, Writing – review & editing.

## References

[1] Scherübl H. Smoking tobacco and cancer risk. Dtsch Med Wochenschr. (2021) 146:412–7. doi: 10.1055/a-1216-7050, PMID: 33735927

[2] Gan H Hou X Zhu Z Xue M Zhang T Huang Z. Smoking: a leading factor for the death of chronic respiratory diseases derived from global burden of disease study 2019. BMC Pulm Med. (2022) 22:149. doi: 10.1186/s12890-022-01944-w, PMID: 35443660 PMC9019969

[3] Gallucci G Tartarone A Lerose R Lalinga AV Capobianco AM. Cardiovascular risk of smoking and benefits of smoking cessation. J Thorac Dis. (2020) 12:3866–76. doi: 10.21037/jtd.2020.02.47, PMID: 32802468 PMC7399440

[4] He H Pan Z Wu J Hu C Bai L Lyu J. Health effects of tobacco at the global, regional, and National Levels: results from the 2019 global burden of disease study. Nicotine Tob Res. (2022) 24:864–70. doi: 10.1093/ntr/ntab265, PMID: 34928373

[5] Alqahtani JS Aldhahir AM Oyelade T Alghamdi SM Almamary AS. Smoking cessation during COVID-19: the top to-do list. NPJ Prim Care Respir Med. (2021) 31:22. doi: 10.1038/s41533-021-00238-8 33958597 PMC8102635

[6] Wachsmann S Nordeman L Billhult A Rembeck G. Tobacco impact on quality of life, a cross-sectional study of smokers, snuff-users and non-users of tobacco. BMC Public Health. (2023) 23:886. doi: 10.1186/s12889-023-15844-z, PMID: 37189128 PMC10184321

[7] Alqahtani JS Aldhahir AM Alanazi Z Alsulami EZ Alsulaimani MA Alqarni AA. Impact of smoking status and nicotine dependence on academic performance of health sciences students. Subst Abus Rehabil. (2023) 14:13–24. doi: 10.2147/SAR.S393062, PMID: 36865699 PMC9970882

[8] Menon PG George S Nair BS Rani A Thennarasu K Jaisoorya TS. Tobacco use among college students across various disciplines in Kerala, India. Tob Use Insights. (2020) 13:1179173x20938773. doi: 10.1177/1179173X20938773 PMC7357023 32699498

[9] Alkhalaf M Suwyadi A AlShamakhi E Oribi H Theyab Z Sumayli I. Determinants and prevalence of tobacco smoking among medical students at Jazan University, Saudi Arabia. J Smok Cessat. (2021) 2021:6632379. doi: 10.1155/2021/6632379 34306226 PMC8279191

[10] Sreeramareddy CT Ramakrishnareddy N Rahman M Mir IA. Prevalence of tobacco use and perceptions of student health professionals about cessation training: results from Global Health professions students survey. BMJ Open. (2018) 8:e017477. doi: 10.1136/bmjopen-2017-017477, PMID: 29804056 PMC5988057

[11] Gibson-Young L Martinasek M Tamulevicius N Fortner M Alanazi AM. Examining electronic nicotine delivery system use and perception of use among college students with and without asthma across the south. J Am Coll Heal. (2022) 70:2026–32. doi: 10.1080/07448481.2020.1842414, PMID: 33151831

[12] Al-Regaiey K Meo S Alobrah M Aljammaz B Alalwan T Alzomia S. Prevalence of electronic and tobacco cigarette smoking among health sciences students in Saudi Arabia. Med Sci. (2022) 26:1–9. doi: 10.54905/disssi/v26i126/ms330e2391

[13] Habib E Helaly M Elshaer A Sriwi D Ahmad MS Mohamed MI. Prevalence and perceptions of e-cigarette use among medical students in a Saudi university. J Family Med Prim Care. (2020) 9:3070–5. doi: 10.4103/jfmpc.jfmpc_235_20, PMID: 32984175 PMC7491770

[14] Bin Abdulrahman KA Alghamdi HA Alfaleh RS Albishri WS Almuslamani WB Alshakrah AM. Smoking habits among college students at a public University in Riyadh, Saudi Arabia. Int J Environ Res Public Health. (2022) 19:11557. doi: 10.3390/ijerph191811557, PMID: 36141829 PMC9517305

[15] Alduraywish SA Aldakheel FM Alsuhaibani OS Jabaan ADB Alballa RS Alrashed AW. Knowledge and attitude toward E-cigarettes among first year university students in Riyadh, Saudi Arabia. Healthcare. (2023) 11:502. doi: 10.3390/healthcare11040502 36833037 PMC9957237

[16] Ahmed LA Verlinden M Alobeidli MA Alahbabi RH AlKatheeri R Saddik B. Patterns of tobacco smoking and nicotine vaping among university students in the United Arab Emirates: a cross-sectional study. Int J Environ Res Public Health. (2021) 18:7652. doi: 10.3390/ijerph18147652, PMID: 34300103 PMC8306162

[17] Alzayani S Hamadeh RR. Tobacco smoking among medical students in the Middle East: identifying areas for intervention. Int J Innov Educ Res. (2015) 3:71–7. doi: 10.31686/ijier.vol3.iss2.314

[18] AlKandari NY. Motivation for smoking in male college students in Kuwait. Int J Health Promot Educ. (2016) 54:217–28. doi: 10.1080/14635240.2016.1157510

[19] Kurdi R Abdul Rahim H Al-Jayyousi G Yaseen M Ali A Mosleh N. Prevalence, risk factors, harm perception, and attitudes toward e-cigarette use among university students in qatar: a cross-sectional study. Front Public Health. (2021) 9:682355. doi: 10.3389/fpubh.2021.682355 34490180 PMC8417713

[20] Rogers CJ Barrington-Trimis JL Unger JB Forster M. Changes in smoking prevalence and perception of smoking on campus before and after a smoke-free university campus policy. J Am Coll Heal. (2022) 70:973–7. doi: 10.1080/07448481.2020.1786097, PMID: 32703095

[21] Jirjees F Dallal Bashi YH Kharaba Z Ahmadi K Barakat M AlObaidi H. Public awareness, prevalence, and regulations for the sale of electronic cigarettes in Arab countries: a narrative review. Tob Induc Dis. (2023) 21:1–17. doi: 10.18332/tid/168435 PMC10603825 37901882

[22] Coleman SRM Piper ME Byron MJ Bold KW. Dual use of combustible cigarettes and E-cigarettes: a narrative review of current evidence. Curr Addict Rep. (2022) 9:353–62. doi: 10.1007/s40429-022-00448-1, PMID: 36467719 PMC9718538

[23] Osei AD Mirbolouk M Orimoloye OA Dzaye O Uddin SMI Benjamin EJ. Association between E-cigarette use and cardiovascular disease among never and current combustible-cigarette smokers. Am J Med. (2019) 132:949–54.e2. doi: 10.1016/j.amjmed.2019.02.016, PMID: 30853474

[24] Mukerjee R Hirschtick JL Arciniega LZ Xie Y Barnes GD Arenberg DA. ENDS, cigarettes, and respiratory illness: longitudinal associations among U.S. youth. Am J Prev Med. (2023) 9:749–3797. doi: 10.1016/j.amepre.2023.12.005, PMID: 38081374

[25] Pisinger C Rasmussen SKB. The health effects of real-world dual use of electronic and conventional cigarettes versus the health effects of exclusive smoking of conventional cigarettes: a systematic review. Int J Environ Res Public Health. (2022) 19:13687. doi: 10.3390/ijerph192013687, PMID: 36294263 PMC9603628

[26] Al-Zalabani AH Aljulifi MZ. Tobacco smoking and type 2 diabetes mellitus in gulf cooperation council countries. Saudi Med J. (2021) 42:1045–6. doi: 10.15537/smj.2021.42.9.1045, PMID: 34470846 PMC9280504

[27] Bennett BL Deiner M Pokhrel P. College anti-smoking policies and student smoking behavior: a review of the literature. Tob Induc Dis. (2017) 15:11. doi: 10.1186/s12971-017-0117-z, PMID: 28163669 PMC5286782

[28] Bardus M El Boukhari N Nakkash R. Development and evaluation of smoke-free or tobacco-free policies in university settings: a systematic scoping review. Health Educ Res. (2020) 35:306–51. doi: 10.1093/her/cyaa009 32702134

[29] Murphy-Hoefer R Griffith R Pederson LL Crossett L Iyer SR Hiller MD. A review of interventions to reduce tobacco use in colleges and universities. Am J Prev Med. (2005) 28:188–200. doi: 10.1016/j.amepre.2004.10.015 15710275

[30] Monshi SS Halpern MT. Factors associated with smoking cessation and smoking cessation interventions in the Gulf cooperation council countries. Saudi Med J. (2019) 40:119–25. doi: 10.15537/smj.2019.2.23904, PMID: 30723855 PMC6402472

[31] Al-Kaabba AF Saeed AA Abdalla AM Hassan HA Mustafa AA. Prevalence and associated factors of cigarette smoking among medical students at king Fahad Medical City in Riyadh of Saudi Arabia. J Family Community Med. (2011) 18:8–12. doi: 10.4103/1319-1683.78631, PMID: 21694953 PMC3114611

[32] Smith DR Leggat PA. An international review of tobacco smoking in the medical profession: 1974-2004. BMC Public Health. (2007) 7:115. doi: 10.1186/1471-2458-7-115, PMID: 17578582 PMC1906758

[33] Monshi SS Collins BN Wu J Alzahrani MAJ Ibrahim JK. Tobacco advertisement, promotion and sponsorship in Arabic media between 2017 and 2019. Health Policy Plan. (2022) 37:990–9. doi: 10.1093/heapol/czac039, PMID: 35668650

[34] Cummings KM Morley C Horan J Steger C Leavell N-R. Marketing to America's youth: evidence from corporate documents. Tob Control. (2002) 11:5–17. doi: 10.1136/tc.11.suppl_1.i5, PMID: 11893810 PMC1766057

[35] Allem J-P Van Valkenburgh SP Donaldson SI Dormanesh A Kelley TC Rosenthal EL. E-cigarette imagery in Netflix scripted television and movies popular among young adults: a content analysis. Addict Behav Rep. (2022) 16:100444. doi: 10.1016/j.abrep.2022.100444 35800212 PMC9253839

[36] Duaso MJ McDermott MS Mujika A Purssell E While A. Do doctors' smoking habits influence their smoking cessation practices? A systematic review and meta-analysis. Addiction. (2014) 109:1811–23. doi: 10.1111/add.12680, PMID: 25041084

