# Supplementary material for: The prevalence and sociodemographic determinants of tobacco and nicotine use among students in healthcare disciplines in Saudi Arabian universities: a cross-sectional survey
Source: Front Public Health. 2024 Mar 7;12:1348370. doi: 10.3389/fpubh.2024.1348370 (PMC10954892; doi:10.3389/fpubh.2024.1348370)
Supplement: Supplementary file 1 [file Data_Sheet_1.docx]

Demographic

1. Age:
2. Gender
3. Male
4. Female
5. Geographical location
6. Central region
7. Eastern region
8. Western region
9. Southern region
10. Northern region
11. Your profession
12. Respiratory Therapy
13. Nursing
14. Medicine
15. Nutrition
16. public health
17. Medical laboratory
18. Emergency medical services
19. Physiotherapy
20. Pharmacy
21. Dentistry
22. Academic level:
23. First year
24. Second year
25. Third year
26. Fourth year
27. Fifth year
28. Sixth year
29. Internship
30. Which of the following substances you have used even for once in the past 30 days?

- Tobacco Cigarettes
- Hookah (Waterpipe, Muʽassel, Jurak)
- Electronic nicotine delivery system (e-cigarettes, e-hookah, vaping)
- Smokeless tobacco (Shammah)
- None of the above
